# Integrated machine learning-based establishment of a prognostic model in multicenter cohorts for acute myeloid leukemia

**DOI:** 10.3389/fonc.2025.1649594

**Published:** 2025-10-14

**Authors:** Lin Yan, Huasong Yu, Xiaoyi Xu, Mingcheng Liu

**Affiliations:** ^1^ State Key Laboratory of Experimental Hematology, National Clinical Research Center for Blood Diseases, Haihe Laboratory of Cell Ecosystem, Institute of Hematology & Blood Diseases Hospital, Chinese Academy of Medical Sciences & Peking Union Medical College, Tianjin, China; ^2^ Department of Oral and Maxillofacial Surgery, Tianjin Union Medical Center, The First Affiliated Hospital of Nankai University, Tianjin, China; ^3^ Tianjin Institutes of Health Science, Tianjin, China

**Keywords:** acute myeloid leukemia, machine learning, prognosis, RNA-Seq, tumor microenvironment

## Abstract

**Background:**

Acute myeloid leukemia (AML) is a highly heterogeneous malignancy, with leukemic cell diversity contributing to disease progression and treatment resistance. This study aimed to evaluate the functional and prognostic significance of leukemic cell-related genes.

**Methods:**

We analyzed single-cell RNA sequencing data to identify malignant marker genes in AML. Consensus clustering was used to assess associations with prognosis and immune responses. A prognostic model, the malignant leukemia marker gene prognostic signature (MLAPS), was developed using 101 models across 10 machine learning algorithms and validated in five independent cohorts. Functional assays were conducted to explore the role of CD69.

**Results:**

We identified a set of malignant marker genes significantly correlated with prognosis and immune classification. The MLAPS showed strong predictive performance, surpassing most clinical features and previously published signatures. Experimental validation confirmed that CD69 promotes malignant progression in AML.

**Conclusion:**

This study highlights the clinical value of leukemic cell-specific genes and presents MLAPS as a robust prognostic tool. CD69 may serve as a potential therapeutic target in AML.

## Introduction

Acute myeloid leukemia (AML) is an aggressive blood cancer, characterized by the abnormal proliferation of immature myeloid cells (1). These cells accumulate in the bone marrow and bloodstream, disrupting the normal production of blood cells. Despite advances in genomic and epigenetic research that have enhanced our understanding of AML, the prognosis remains poor, particularly in older patients (1, 2). AML prognosis varies significantly depending on several factors, including the patient’s age, overall health, genetic mutations, and response to initial therapy. Older patients or those with adverse genetic mutations tend to have worse outcomes. Even with intensive treatment, the 5-year survival rate of <30% (3). New treatment combinations, such as targeted therapies and immunotherapies, are being explored to improve survival rates (4). In conclusion, while there have been some advances in treating AML, significant challenges remain. In the era of precision medicine, personalized innovative therapies and the development of effective survival models may be key to improving prognosis.

An ideal prognostic biomarker should demonstrate stable and consistent expression across different AML patients, especially within the various subtypes of the French-American-British (FAB) classification. A biomarker that exhibits homogeneous expression across subtypes can more effectively predict disease progression and treatment outcomes. However, due to the high heterogeneity of AML, single-gene biomarkers often fail to accurately predict disease prognosis in all patients. Thus, a multi-gene prognostic model has been considered a potential strategy to address the issue of AML heterogeneity effectively. In recent years, with the rapid advancements in molecular biology, genomics, and bioinformatics, the gene expression characteristics of AML have been explored in greater depth. Through large-scale data analysis, researchers have identified a series of genes associated with AML prognosis (4, 5). These gene signatures have not only been validated in the laboratory but are also regarded as potential prognostic biomarkers and therapeutic targets for AML. Multi-gene models can integrate various gene information, revealing individualized pathological characteristics of AML patients, thereby providing more precise references for clinical decision-making. However, despite the promising potential of multi-gene prognostic models, their application in clinical practice still faces numerous difficulties and challenges. First, existing AML transcriptome data have not been fully utilized. Inappropriate application of machine learning methods, coupled with a lack of rigorous validation across different cohorts, has limited the ability to fully explore potential biomarkers, restricting the accuracy and clinical applicability of these models. In summary, while multi-gene prognostic models theoretically hold promise as effective tools for addressing AML heterogeneity, there remain significant challenges in model development, data utilization, and cross-cohort validation.

To develop an ideal prognostic model, we identified malignant cell marker genes in AML datasets by single-cell RNA sequencing (scRNA-seq). Through in-depth analysis of its prognosis, immune infiltration, and clinical features using 101 integrated machine learning-based models, we developed and validated a machine learning-based AML gene prognostic signature (MLAPS) across multiple cohorts, which may help optimize precision treatment and further improve the clinical outcomes of AML patients.

## Materials and methods

### Data collection and processing

We acquired the 6 independent public datasets in this work, they were collected from multiple repositories including The Cancer Genome Atlas (TCGA, https://portal.gdc.cancer.gov/), Gene Expression Omnibus (GEO, https://www.ncbi.nlm.nih.gov/geo/), and Therapeutically Applicable Research To Generate Effective Treatments (TARGET, https://www.cancer.gov/ccg/research/genome-sequencing/target). Among the datasets, the RNA-seq data was transformed into transcripts per kilobase million (TPM), log2 transformed, and further removed batch effects. Finally, we enrolled four datasets with completive OS information, TCGA-LAML (n = 132), GSE37642 (n = 417), GSE12417 (n = 163), and GSE106291 (n = 250) were used for construction and validation of our prognostic model. The independent dataset GSE10358 (n = 91) was used to verify the predicting value of MLAPS, and the acute lymphoid leukemia dataset TARGET-ALL (n = 611) was used to assess the applicability of our signature in other blood tumors.

### Identification of malignant cell marker genes

We downloaded the AML scRNA-seq dataset GSE116256 from GEO, raw gene expression matrices were imported and processed using the Seurat R package (6), and we set the parameters to remove low-quality cells: min.cells = 3, nFeature_RNA > 50, percent.mt < 15. We got a single-cell object and then standardized the data via NormalizeData function. Then we performed FindVariableGenes function to calculate high variable genes, and conducted ScaleData and RunPCA to standardize the data and principal component analysis (PCA) analysis, the clusters were found using FindClusters function (dims.use = 1:30, resolution = 0.5). We conducted uniform manifold approximation and projection (UMAP) analysis for dimension reduction and visualization of marker gene expression. Cell types were annotated based on the expression of marker genes. To identify marker genes for each cell type, we employed the FindAllMarkers function implemented in the Seurat package. This function was used in conjunction with the Wilcoxon–Mann–Whitney test to assess differential gene expression between a given cell type and all other cell types. To ensure the robustness of marker selection, we applied the following filtering criteria: (i) genes had to be expressed in at least 10% of the cells within the tested cluster, (ii) only genes with an adjusted P-value < 0.05 were retained, and (iii) genes were further restricted to those with an absolute log fold change (|logFC|) > 1. Genes meeting these thresholds were designated as marker genes for subsequent analyses. Additionally, the CellChat package (7) was utilized to identify over-expressed interactions in the malignant bone marrow microenvironment.

### Consensus clustering

Based on the clustering of malignant cells marker gene profiles, we utilized ConsensusClusterPlus R package (8) to classify patients in the meta-cohort (TCGA-LAML, GSE37642, GSE12417, GSE106291, n = 962) into different subtypes. The optimal number of clusters was determined using the cumulative distribution function (CDF) and the relative change in the area under the CDF curve, resulting in the construction of a consensus matrix.

### Uni-Cox regression analysis

We utilized intersection genes to conduct univariate Cox regression analysis in the four cohorts for building a prognostic model. The consensus prognosis genes were filtered according to the following criteria: P-value < 0.05 and | HR| >1.

### Machine learning−based prognostic model construction and validation

The 10 basic machine learning algorithms performed in this research were CoxBoost (Boosting in Cox regression), Lasso (Least Absolute Shrinkage and Selection Operator), Ridge, plsRcox (partial least squares regression for Cox), RSF (random survival forest), SuperPC (supervised principal components), GBM (generalized boosted regression modeling), stepwise Cox, Enet (elastic network), survival-SVM (survival support vector machine). Additionally, several of the algorithms, including Lasso, RSF, stepwise Cox, and CoxBoost, incorporated feature selection functions. The 10 machine learning algorithms were integrated into 101 model combinations, Harrell’s concordance index (C-index) was calculated for each model across all validation datasets. TCGA-LAML, GSE37642, GSE12417, and GSE106291 were each split into training and test cohorts at a 3:1 ratio and used for constructing the optimal model. With the optimal model Machine learning-based AML prognosis signature (MLAPS), patients were classified into high-risk and low-risk groups according to the median risk scores obtained from the four independent validation cohorts. The prognostic significance and predictive performance of the optimal model were evaluated through receiver operating characteristic (ROC) curves and Kaplan–Meier (KM) survival curves. Moreover, GSE10358 and TARGET-ALL were used as independent validation cohorts to further assess the accuracy of MLAPS.

### GO, KEGG, GSEA

In this study, we used clusterProfiler R package for enrichment analysis (9). Gene Ontology (GO) analysis is widely used to annotate various genes and shed light on their associated functions. Additionally, the Kyoto Encyclopedia of Genes and Genomes (KEGG) database is a valuable tool for integrating genomic data, aiding in the examination of gene functions and expression patterns. To pinpoint statistically significant gene sets, Gene Set Enrichment Analysis (GSEA) was performed. In this analysis, a threshold of P < 0.05 was set to determine statistical significance for GO, KEGG, and GSEA results.

### Tumor immune microenvironment

Single-sample gene set enrichment analysis (ssGSEA) implemented in GSVA package was utilized to quantify the relative infiltration of 28 immune cell types in the Meta cohort (TCGA-LAML, GSE37642, GSE12417, GSE106291, and GSE10358). ESTIMATE algorithm was performed to verify the tumor immune microenvironment results.

### Data availability statement

The original data supporting the findings of this study are available within the article. Any additional questions or requests for further clarification on the data can be addressed by contacting the corresponding authors. The public data utilized in this study can be accessed through the TCGA Research Network portal (https://portal.gdc.cancer.gov/) and Gene Expression Omnibus (GEO, https://www.ncbi.nlm.nih.gov/geo/).

### Cell culture and infection

K562 cells and Kasumi-1 cells were maintained in RPMI-1640 medium supplemented with 20% fetal bovine serum (FBS)(GIBCO)at 37°C and 5%CO2. Lentiviral particle was produced in HEK293T cells by using psPAX2 and pMD2.G helper vectors. We used lentivirus containing CD69-specific interference sequences or scramble fragments to infect with AML cell lines K562 and Kasumi-1. After 48 hours of infection, GFP+ cells were sorted by flow cytometry. The cell lines used in this experiment were gifted by Professor Jianxiang Wang’s laboratory.

### Western blot

Lysates were prepared using RIPA lysis buffer (Beyotime, Shanghai, China) with a proteinase inhibitor (Roche, Basel, Switzerland). After lysis on ice for 30 mins, the supernatant was collected. Antibodies were used as follows: CD69 Rabbit pAb (ABclonal, A2045, 1:1000, Wuhan, China), GAPDH Mouse mAb (ABclonal, AC002, 1:1000, Wuhan, China).

### RNA extraction and RT-qPCR

RNA extraction was performed using TRIzol Reagent (Invitrogen, USA) following the manufacturer’s instructions. RT–qPCR was performed using a HiScript III 1st Strand cDNA Synthesis Kit (Vazyme, China) and SYBR^®^Green Realtime PCR Master Mix (TOYOBO, JAPAN) according to the manufacturer’s instructions. GAPDH was used as the endogenous control.

### Cell proliferation assay

Cell proliferation was measured using Cell Counting Kit (CCK-8) (Beyotime, Shanghai, China).2x10^3^ cells were seeded into each well of a 96-well plate, transfected with the specified siRNA, and incubated for 1d, 2d, 3d, 4d, 5d, 6d, 7d and 8d respectively. Two hours before the end of the incubation, 10 μl CCK-8 reagent was added to each well. The optical density (OD) at 450 nm (OD450nm) was measured using a microplate reader.

Resuspend 1x10^3 cells in 300 μl culture medium MethoCult™ H4535 (STEMCELL, Canada) and seed them into a 24-well plate. The colony number was counted 14 days after plating.

5-Ethynyl-2′-deoxyuridine (EdU) analysis was performed using the BeyoClick™ EdU Cell Proliferation Kit with Alexa Fluor 594(Beyotime, Shanghai, China) according to the manufacturer’s instruction.

### Flow cytometric analysis

Cell apoptosis was assessed with flow cytometry Cell cycle stage was assessed with flow cytometry and measured DNA content after incubation with RNase A and propidium iodide (PI). Apoptosis was performed by using annexin V-FITC/PI Apoptosis Detection Kit (Yeasen Biotech). For differentiated cells assay, cells were incubated on ice for 30min in the dark with antibodies as follows: PE-anti-human CD11b (Biolegend, USA), APC-anti-human CD14 (Biolegend, USA).

### Murine MLL AF9 leukemia model construction and treatment

A transplantable MLL-AF9-inducible murine AML model was generated as previously reported (10). In brief, Lin- cells were isolated from the bone marrow (BM) of 8-week-old wild-type (WT) C57BL/6 mice and transduced with MSCV-MLL-AF9-IRES-GFP retrovirus. A total of 5x10^5 infected cells were then transplanted into sublethally irradiated (4.5 Gy) C57BL/6 recipient mice via tail vein injection. On day 7 post-transplantation, the mice were randomly assigned into two groups, with one group receiving 4 mg/kg of BTK-IN-8 and the other PBS, administered every other day for 12 days. All animals were euthanized on day 19, and leukemia burden in peripheral blood, spleen, and BM was evaluated by flow cytometry (FACS). All the mouse experiments were approved (the reference number is IHCAMS-DWLL-NSFC2024126-1) by the IACUC of the Institute of Haematology, Chinese Academy of Medical Science.

### Statistical analysis

All data preprocessing, analysis, and result visualization in this study were carried out using R version 4.3.0 or Prism9. Continuous variables were evaluated using either the Wilcoxon rank-sum test or Student’s t-test. Categorical variables were analyzed statistically using either the Chi-square test or Fisher’s exact test. Univariate and multivariate Cox regression, along with Kaplan–Meier survival analysis, were conducted using the survival package. The timeROC package was applied to generate both ROC and calibration curves. P < 0.05 was considered statistically significant. Wet experiments, including WB, cell migration, colony formation, and EdU cell proliferation assays, have been performed in triplicate.

## Results

### Identification of AML cell marker genes by scRNA−seq

**Figure 1:**
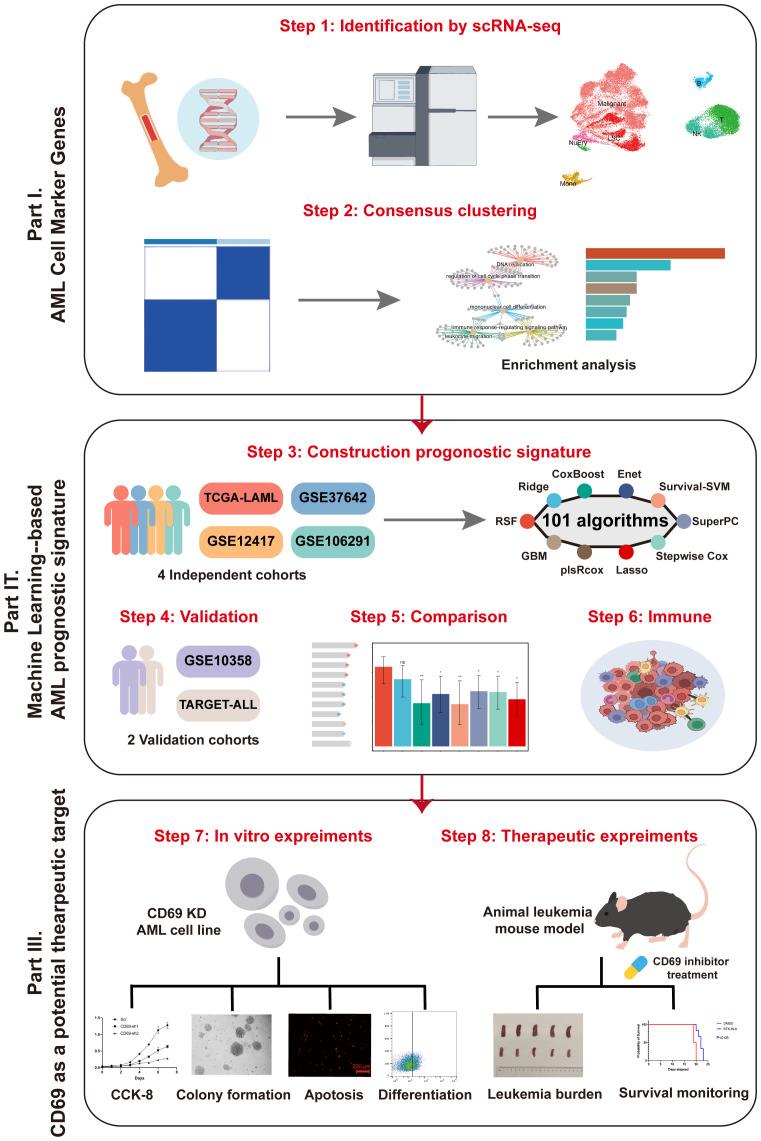
The workflow of our research.

**Figure 2:**
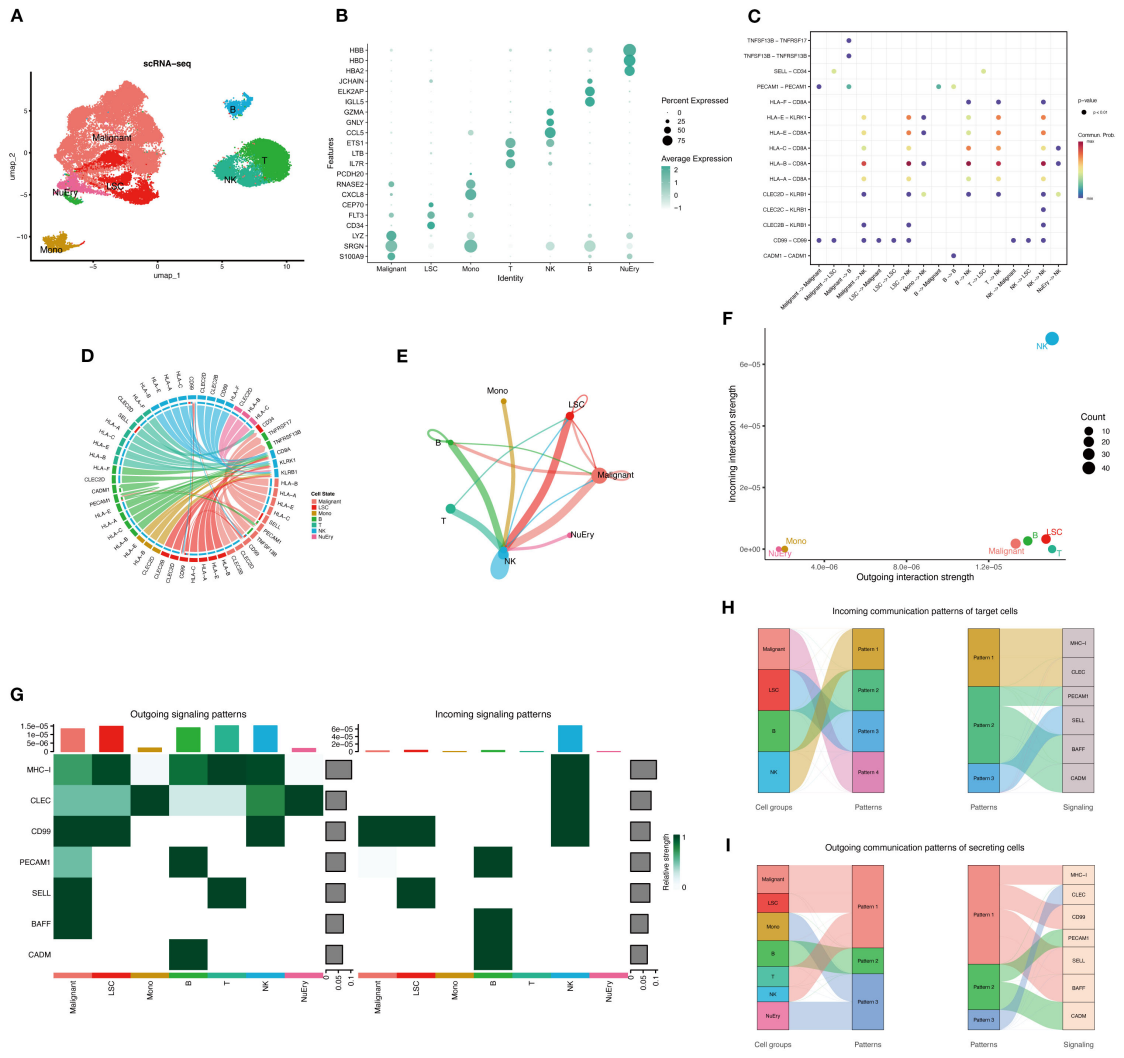
The overview of the AML bone marrow microenvironment. **(A)** UMAP plot shows the cell type identification of filtered high-quality cells from AML patients’ bone marrow samples. Data from GSE116256. **(B)** Bubble plot illustrates the average expression levels and the percentage of cells expressing the top DEGs in each cell cluster. **(C)** Cellchat bubble plot illustrated all the cell communication pathways. **(D)** Chord plot shows the inferred intercellular communication network in the AML bone marrow microenvironment. **(E)** Circle plot presents the interaction number between each cell type. The line width represented the interaction number. **(F, G)** NK cells are active in both sending and receiving signals, while LSCs, malignant cells, and other immune cells are only active in signal sending. Normal myeloid and erythroid cells are not active in cell communication. **(H, I)** The river plot illustrates the correspondence between cell types and patterns, as well as the patterns’ associated signaling molecules.

The research graphic workflow is displayed in **Figure 1**. We downloaded the scRNA-seq dataset from the GEO database (GSE116256) (11), which includes 36 AML samples. We retained 31,815 cells for further downstream analysis after quality control. We conducted dimensionality reduction and clustering using UMAP (**Figure 2A**). The cells were classified into 10 distinct cell types (**Figure 2B**, **Supplementary 1A, B**), each identified by validated marker genes: malignant cells were marked with S100A9 and SRGN; LSCs were marked with FLT3 and CEP70; Monocytes were marked with SRGN and CXCL8; T cells were marked with IL7R and EST1; NK cells were marked with CCL5 and GNLY; B cells were marked with IGLL5 and JCHAIN; NuEry were marked with HBA2 and HBB. Through cell-cell communication analysis, we identified the most prominent receptor-ligand pairs (P < 0.01) among various cell types within the AML bone marrow microenvironment (**Figures 2C-E**). LSC cells exhibited cell-cell communication with NK and T cells (Sup 1.C), and NK cells showed the highest signal strength in both outgoing and incoming communication (**Figures 2F, G**). We categorized the incoming signals of target cells into four patterns, while the outgoing signals of secreting cells were divided into three patterns (Sup 1.D-E), identifying pattern-associated signals (**Figures 2H, I**). Pattern-related signals play an important role in immune responses, such as MHC-I and CD99 (12, 13). Through the above analysis, we identified the marker genes of AML cells (Malignant and LSC) and concluded that the infiltration of immune cells plays a crucial role in shaping the AML bone marrow microenvironment.

### Consensus clustering based on AML marker genes and their relationship with prognosis

**Figure 3:**
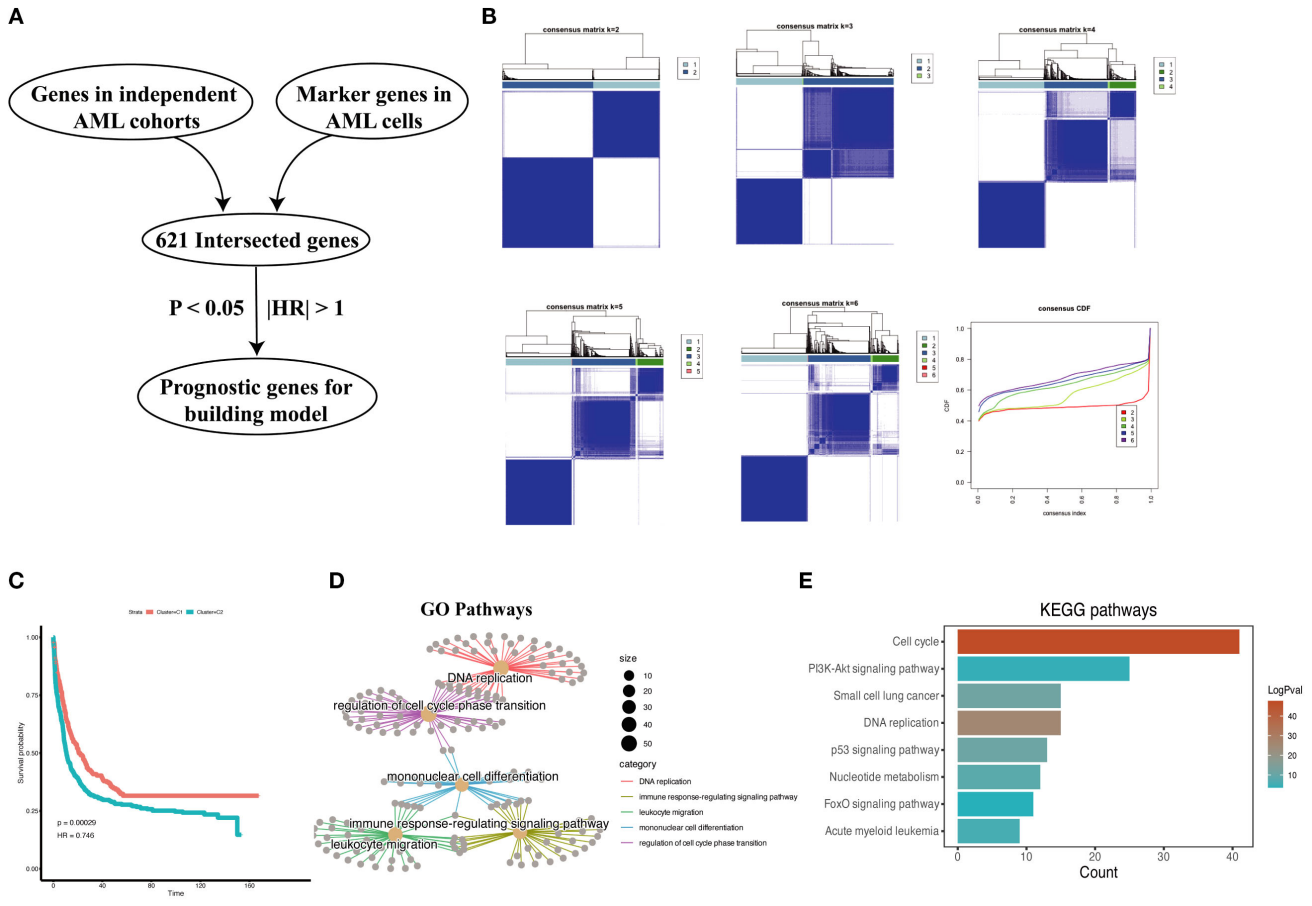
Prognostic association of malignant marker genes. **(A)** The process of selecting prognostic genes for the constructing model. **(B)** Consensus clustering analysis based on malignant marker genes from AML scRNA-seq dataset, samples from four bulk RNA-seq datasets (TCGA-AML, GSE106291, GSE12417, GSE37642, n = 962) are divided into subgroups. The CDF curves of consensus matrix for each k, the clustering results are optimal when k=2. **(C)** Kaplan–Meier curves for overall survival (OS) in C1 and C2. **(D)** Cnetplot of GO pathways derived from highly expressed DEGs in C2 group shows enrichment in pathways. **(E)** Bar plot of KEGG pathways derived from highly expressed DEGs in C2 group indicates a significant association between these genes and cell proliferation functions.

We obtained 621 intersected marker genes by intersecting the marker gene of AML in scRNA-seq and the gene in four independent cohorts, we conducted consensus clustering (8) on AML samples from four independent cohorts (TCGA-LAML, GSE37642, GSE12417, GSE106291) with the marker gene set, testing cluster numbers (k) ranging from 2 to 6 (**Figures 3A, B**). Based on the cumulative distribution function (CDF) curves derived from the consensus score matrix heatmap, the optimal number of clusters was determined to be k = 2 (**Figure 3B**). A total of 415 patients were assigned to the C1 group, while 566 patients were assigned to the C2 group, the survival analysis demonstrated that the C2 group had a worse prognosis compared to the C1 group (**Figure 3C**). We performed GO and KEGG enrichment analyses on the differentially expressed genes that were upregulated in the C2 group compared to the C1 group. The results showed that pathways related to DNA replication and the cell cycle were enriched in the C2 group, with significant upregulation of proliferation and metabolism-related pathways, such as the p53 and PI3K/Akt pathways (**Figures 3D, E**), and the above findings suggested that patients with stronger proliferative capacity of AML cells tend to have a poorer prognosis (14, 15). Additionally, the enrichment of immune response-regulating signaling pathway indicates that the tumor immune microenvironment plays a critical role in AML patients. However, the influence of immune responses within the AML bone marrow microenvironment on tumor promotion or suppression requires further analysis. Subsequently, we performed a preliminary univariate Cox regression analysis (P < 0.05, |HR| > 1), which identified prognosis-related genes for constructing a machine learning signature (**Figure 3A**). Our signature genes underwent several rounds of screening, and the optimal model was chosen from 101 algorithms. This approach produced the Machine learning-based AML prognosis signature (MLAPS) with better extrapolation potential, ensuring reliable performance across different AML patient populations and enhancing its clinical relevance.

### Integrative construction of an AML prognostic signature based on machine learning

**Figure 4:**
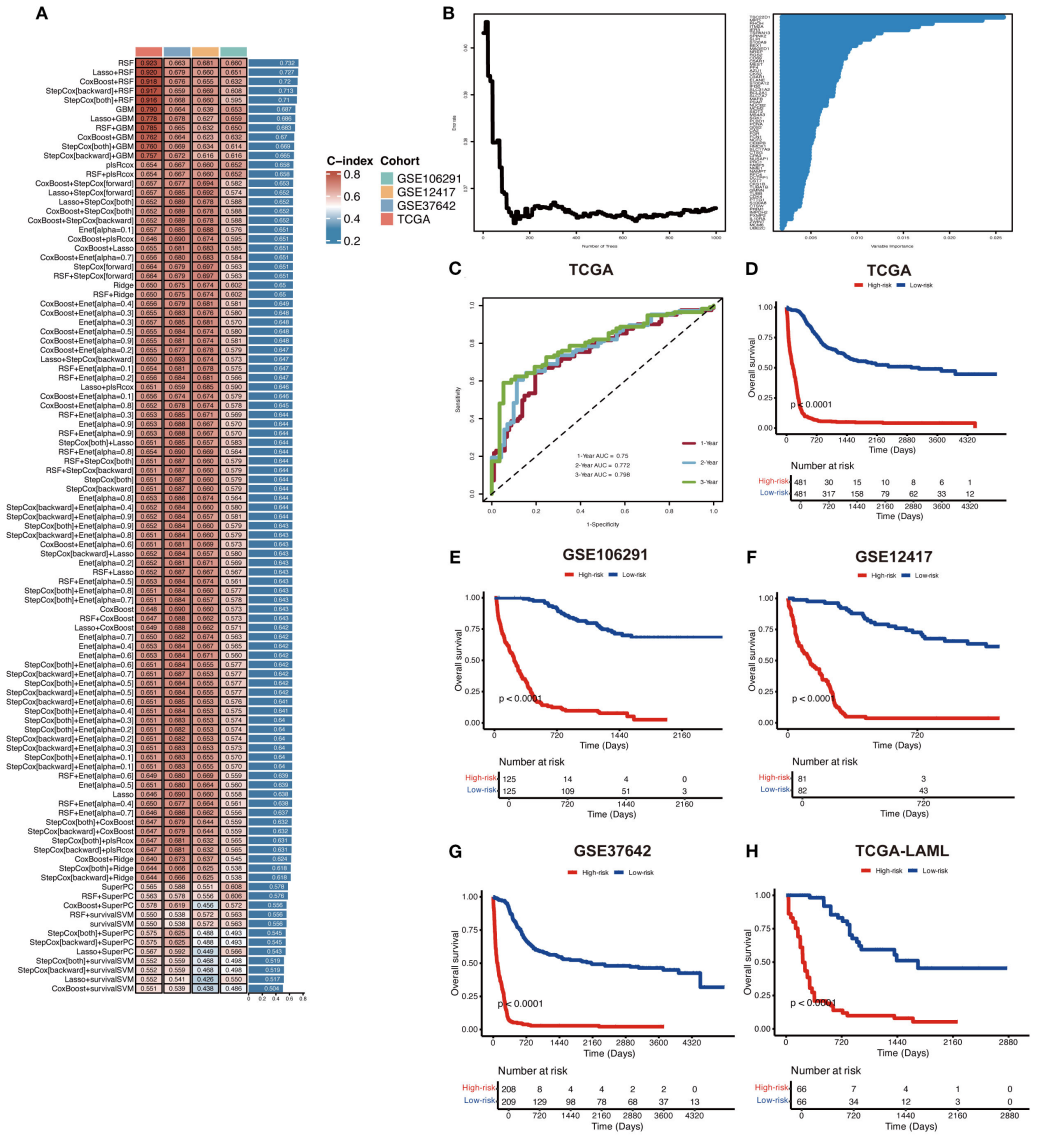
Construction of a prognostic model for AML via machine learning-based integrative procedure. **(A)** In total, 101 predictive models were constructed using 10 basic algorithms, and the C-index of each model was further calculated across all cohorts. **(B)** The importance of prognostic genes in the RSF model. **(C)** ROC curves present 1-, 2-, and 3-year OS in the test set of the meta cohort. **(D-H)** KM curves of OS in the meta, GSE106291, GSE12417, GSE37642, and TCGA-AML cohorts based on the model showed longer survival time in low-risk groups.

To develop an AML prognostic signature, we used 101 machine learning models, including 10 basic algorithms: CoxBoost, Lasso, Ridge, plsRcox, RSF, SuperPC, GBM, stepwise Cox, Enet, survival-SVM (16, 17). The meta cohort (the four independent AML cohorts mentioned above) with the filtered prognostic genes was subjected to our machine learning-based integrative procedure to develop an AML prognostic signature (MLAPS). We applied the LOOCV (Leave One Out Cross Validation) framework to fit 101 different prediction models and computed the C-index for each model across all validation cohorts. Notably, the most optimal model was RSF with the highest average C-index (0.732), and the model achieved the leading C-index across all validation cohorts (**Figure 4A**). As the number of survival trees increased, the error rate curve eventually plateaued, indicating that the number of trees in the RSF model was appropriate and sufficient for accurate predictions (**Figure 4B**). This stabilization of the error rate suggests that adding more trees would not significantly improve the model’s performance. Additionally, we presented the importance of each prognostic gene within the model, demonstrating how individual genes contribute to the overall survival prediction (**Figure 4B**). By evaluating the variable importance of these genes, we can gain insights into the key factors driving survival outcomes in AML patients. ROC analysis was performed to evaluate the discriminative ability of the MLAPS model in predicting patient survival outcomes. The area under the curve (AUC) values for 1-, 2-, and 3-year survival predictions were 0.75, 0.772, and 0.798, respectively, in the meta cohort (**Figure 4C**). These AUC values indicate that the MLAPS model demonstrates strong predictive performance over time, with increasing accuracy as the prediction horizon extends. Using the median MLAPS score as the cutoff value, all patients were stratified into high-risk and low-risk groups. As shown in **Figures 4D-H**, patients in the high-risk group exhibited significantly worse overall survival (OS) compared to those in the low-risk group across the meta cohort and all four individual cohorts (P < 0.05 in each case). These findings suggest that the MLAPS model is a robust predictor of patient prognosis, effectively differentiating between individuals with higher and lower survival probabilities. The clear survival disparity between the two risk groups underscores the utility of MLAPS in risk stratification and its potential application in clinical decision-making.

### Validation of the MLAPS model

**Figure 5:**
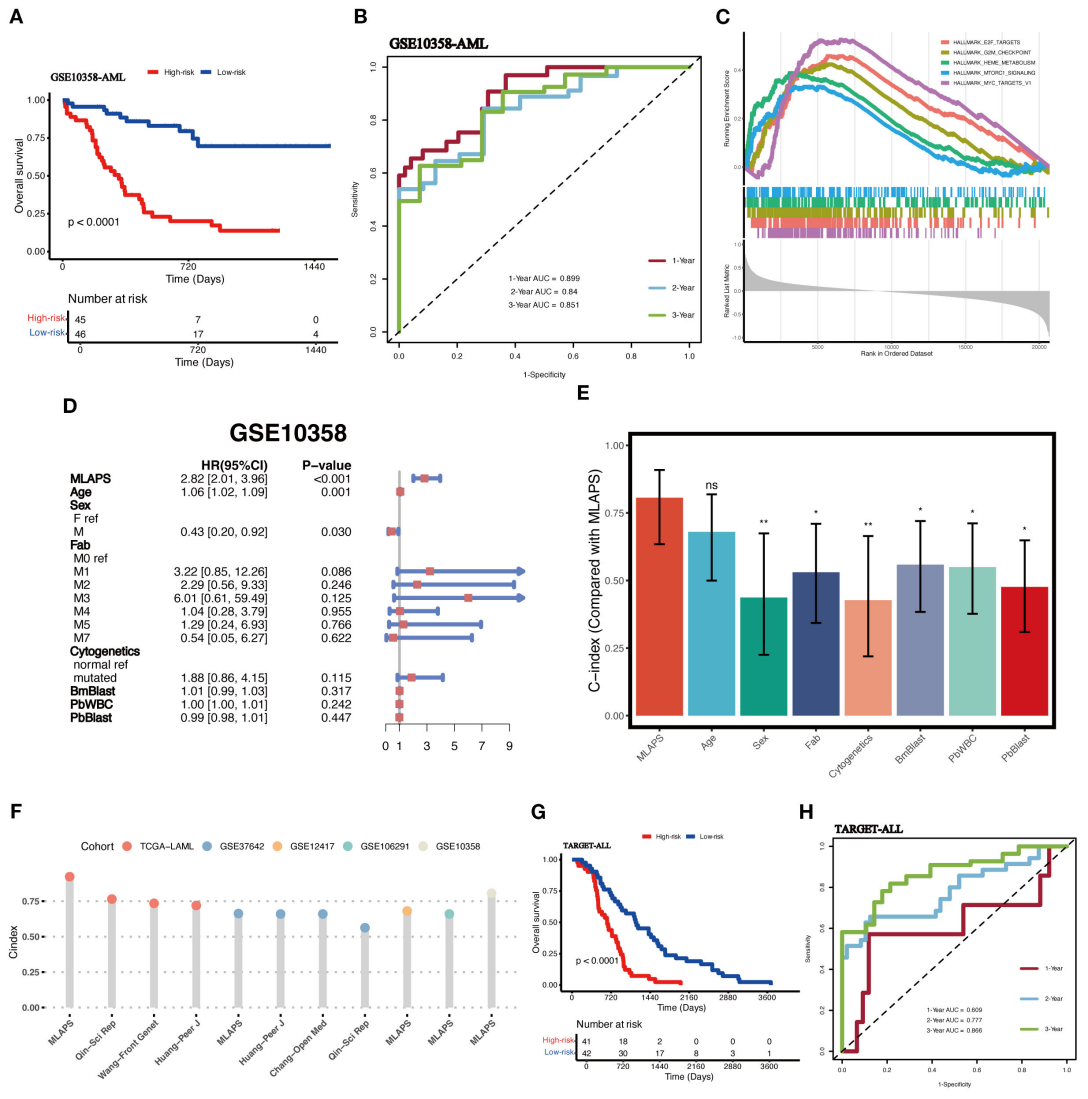
Validation in independent acute leukemia cohorts. **(A)** KM curves of OS in the AML validation cohort (GSE10358) based on the model showed longer survival time in low-risk groups. **(B)** ROC curves present 1-, 2-, and 3-year OS in the AML validation cohort (GSE10358). **(C)** The top 5 Hallmark-enriched pathways in the High MLAPS groups. **(D)** Multivariable Cox regression analysis of OS in GSE10358. Statistic test: two-sided Wald test. Data are presented as hazard ratio (HR) ± 95% confidence interval (CI). **(E)** The predictive performance of MLAPS was compared to common clinical and molecular variables in the GSE10358 dataset. *P < 0.05; **P < 0.01. **(F)** Comparison between MLAPS and previously published signatures. **(G)** KM curves of OS in the ALL validation cohort (TARGET-ALL). **(H)** ROC curves present 1-, 2-, and 3-year OS in the ALL validation cohort (TARGET-ALL) and the predict value in other blood cancers.

To further validate the performance of the MLAPS model in our study and to mitigate the risk of overfitting in the prognostic model, we employed an independent dataset (GSE10358, n = 91) as the validation cohort. This external validation step is critical for assessing the generalizability of the MLAPS model beyond the meta cohort. The KM analysis for OS demonstrated that the high MLAPS group possessed significantly shorter survival in the AML validation cohort (**Figure 5A**). the ROC curves were plotted and the AUC was calculated at 1-, 2-, and 3-years were 0.899, 0.84, 0.851 (**Figure 5B**). GSEA was applied to elucidate the potential functional pathways of MLAPS in the high-score group of meta and validation cohorts. As illustrated in **Figure 5C**, the high MLAPS group was remarkably enriched for proliferate and metabolism-related pathways, such as E2F transcription factor targets, MYC targets, and mTORC1 signaling, which partly explained its more advanced grades and worse prognosis (18, 19). Additionally, to further assess the predictive performance of MLAPS, we used the TARGET-ALLP2 dataset (n = 83) as another validation cohort to investigate its predictive capability in lymphoid leukemia. KM analysis indicated that the high MLAPS group also exhibited shorter overall survival in ALL (acute lymphoblastic leukemia) (**Figure 5G**). ROC analysis measured the discrimination of MLAPS, with 1-, 2-, and 3-year AUCs of 0.609, 0.777, and 0.866 (**Figure 5H**). By testing the model on the separate datasets, we can ensure its predictive accuracy holds across different patient populations, reinforcing the robustness and reliability of MLAPS as a prognostic tool for AML.

### Evaluation of the MLAPS model

To assess the prognostic performance of MLAPS, we included the AML validation cohort for both univariate and multivariate Cox regression analyses. The results demonstrated that MLAPS is an independent risk factor for OS, with a hazard ratio (HR) of 2.82 (95% CI: 2.01–3.96, P < 0.01, **Figure 5D**). This indicates that patients with higher MLAPS scores are at significantly increased risk of poor survival outcomes, independent of other clinical variables. In addition, we calculated the C-index for MLAPS, along with other clinical factors including Age, Sex, FAB classification, Cytogenetics, BM Blast count, PB WBC count, and PB Blast count (**Figure 5E**). Among these, MLAPS demonstrated the highest C-index at 0.806 (95% CI: 0.634–0.908), indicating its superior predictive accuracy for overall survival. Comparisons between MLAPS and other clinical variables revealed statistically significant differences, with P-values less than 0.05 in each case. These results highlight the robust prognostic power of MLAPS compared to traditional clinical indicators in AML. We conducted a comparison of gene expression-based prognostic signatures in AML (20–23) (**Figure 5F**), and while most models performed well within their respective training datasets, they lacked validation in independent external cohorts. This limitation is likely due to the reduced generalizability of models that are overfitted to their training data (23). This underscores the importance of validating prognostic models in external cohorts to ensure their broader applicability and reliability in clinical settings.

### The immune landscape and molecular mechanisms of MLAPS

**Figure 6:**
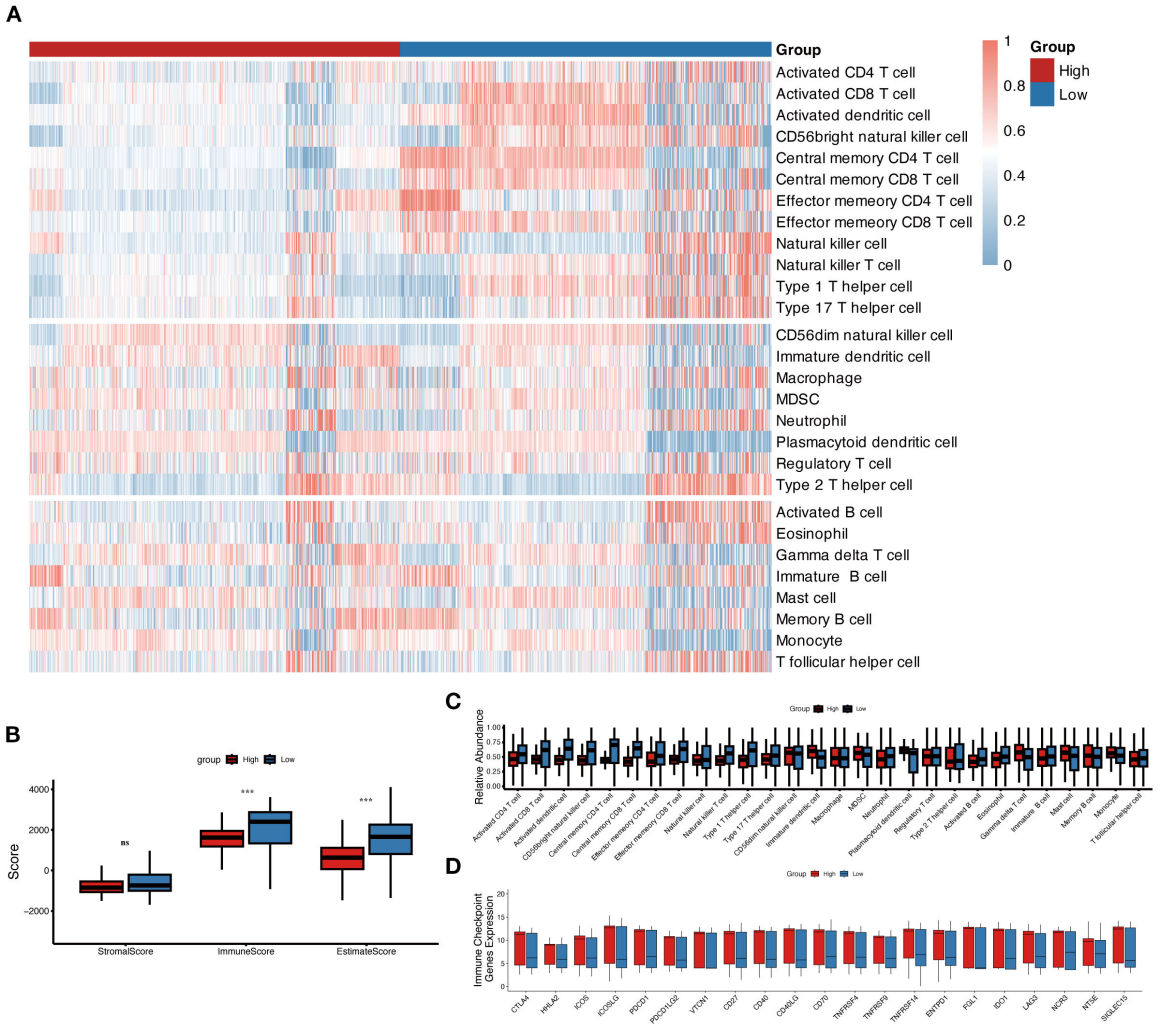
The immune landscape in the high and low MLAPS groups. **(A)** Heatmap of 28 immune cell types in the high and low MLAPS groups. The 12 cell types above are anti-cancer types, and the 8 cell types in the middle are pro-cancer types. **(B)** Boxplots compare the ESTIMATE score, stromal score, immune score, and tumor purity between high and low MLAPS groups. **(C)** Boxplots present the relative abundance of each cell type. The gene order of the boxplots is the same as the heatmap. **(D)** Boxplots display the relative expression levels of 21 immune checkpoint profiles between the high and low MLAPS groups.

In the above GO and KEGG enrichment analyses, immune response-related pathways were enriched in the high-risk group (**Figures 3D, E**). To further explore this, we investigated the immune landscape and expression of immune checkpoint molecules (ICMs) between the high and low MLAPS groups across five cohorts (GSE106291, GSE12417, GSE37642, TCGA-AML, and GSE10358). Using single-sample gene set enrichment analysis (ssGSEA), we observed that the low MLAPS group exhibited significantly higher infiltration of anti-tumor immune cells, including activated T cells, central memory T cells, effector memory T cells, activated dendritic cells, and natural killer cells (all P < 0.05, **Figures 6A, C**). According to the ESTIMATE algorithm, aside from stromal score, the low and high MLAPS groups showed significant differences in both Immune Score and Estimate Score (P < 0.01, **Figure 6B**). Furthermore, analysis of 21 ICMs revealed that the high MLAPS group had markedly elevated expression levels of key immune checkpoint molecules, such as CTLA4, HHLA2, ICOS, PDCD1LG2, VTCN1, CD40, and CD70 (**Figure 6D**). Together, these findings suggest that the high MLAPS group is more likely to benefit from immunotherapy, and targeting immune-related factors may serve as an effective therapeutic strategy to improve the prognosis of high-risk AML patients.

### CD69 promotes the malignant progression of AML cells

**Figure 7:**
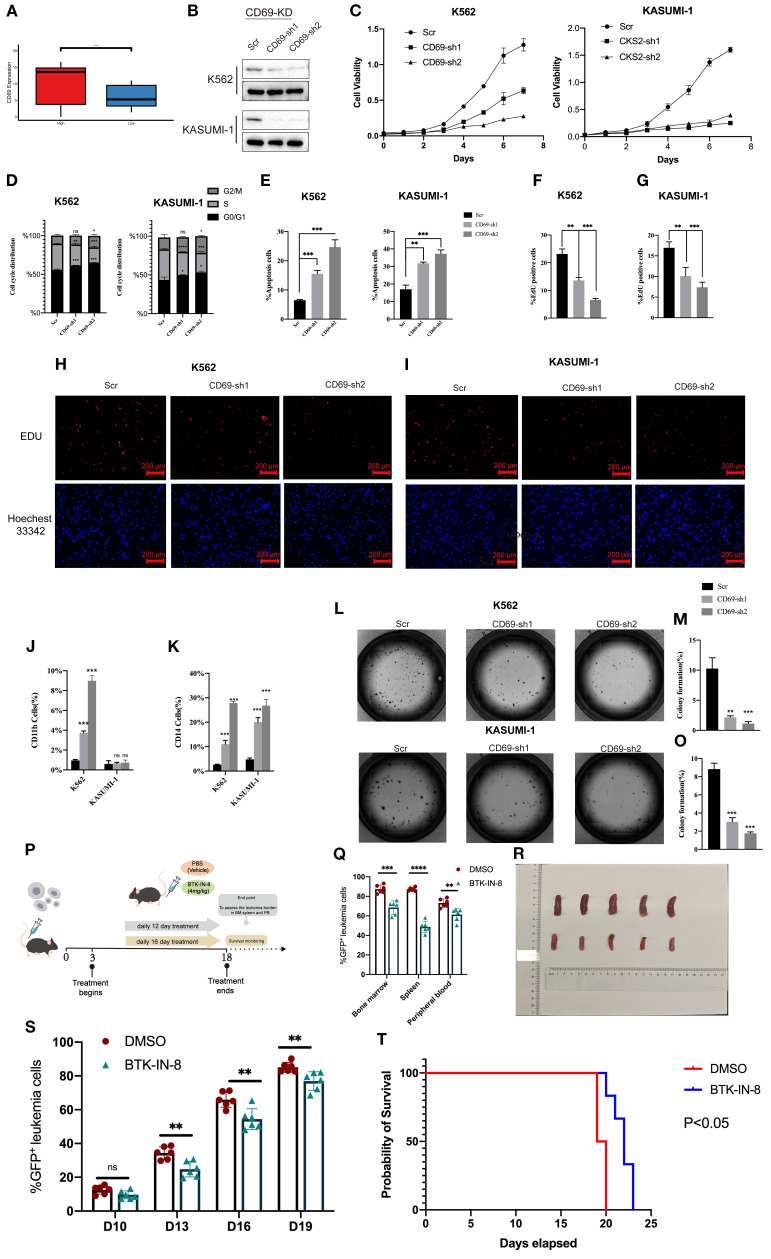
CD69 promotes malignant proliferation and apoptotic escape of AML cells. **(A)** Boxplot compares CD69 expression of the high and low MLAPS groups. **(B)** Western blot and qPCR showed CD69 KD efficiency in K562 and KASUMI-1 cells after transduction with shRNA lentiviruses targeting CD69. **(C)** In the CCK-8 assay, the cell viability of sh-CD69 AML cells was diminished. **(D)** Cell cycle analysis revealed that the proportion of G2/M phase cells significantly increased in sh-CD69 AML cells. **(E)** Flow cytometric analysis of apoptosis frequency of AML cells expressed Scramble shRNA (Scr), sh-CD69.Annexin V1 indicated the apoptotic cells. **(F-I)**. In the EdU experiment indicated a reduction in the ability to proliferate after sh-CD69 treatment. ***P < 0.01.***P < 0.001. **(J, K)** Flow cytometric analysis of differentiated cell frequency (CD11b and CD14 cells ratio) of, K562 and KASUMI-1 cells expressed Scramble shRNA (Scr). ***P < 0.001. **(L-O)** Colony formation assay assessing the colony-forming ability of K562 and KASUMI-1 cells. **(P)** *In vivo* efficacy estimation of BTK-IN-8 treatment. MLL-AF9 leukemia mouse models 2 arms of treatment were applied as vehicle, BTK-IN-8 4mg/kg daily. Leukemia burden in bone marrow, spleen, and peripheral blood at each observational timepoint and overall survival were observed and compared among arms respectively. **(Q)** The percentage of GFP-positive leukemia cells in the bone marrow, spleen, and peripheral blood of the Scr group and the drug treatment group. **(R)** The spleen size of AML mice in two groups (Top: Scr group, Bottom: BTK-IN-8 group). **(S)** The percentage of GFP-positive leukemia cells at D10, D13, D16, and D19 in the Scr group and the drug treatment group. **(T)** The survival curves of AML mice in the two groups. **P<0.01.

AML is a malignant hematologic tumor characterized by the abnormal proliferation of immature myeloid cells in the bone marrow and peripheral blood (1). Therefore, we hypothesized that the upregulated genes in our model are valuable for understanding the malignant progression of AML cells. Consistent with our expectations, an extensive literature review revealed that most of these upregulated genes play critical roles in AML progression and prognosis. CD69 has been identified as a biomarker that promotes the malignant progression of various cancers (24) and is highly expressed in AML (25). CD69 demonstrated a relatively high variable importance within the prognostic model (**Figure 4B**). In the AML cohort, CD69 expression was positively correlated with the immune microenvironment abundance of pro-cancer immune cells, including CD56^dim^ natural killer cells, immature dendritic cells, and MDSCs (**Supplementary Figure S2**). Additionally, CD69 was highly expressed in the high MLAPS group across five combined AML cohorts (**Figure 7A**). However, despite its recognized overexpression, the precise function of CD69 in AML remains elusive. Further research is required to elucidate its specific role in the disease, which may provide new insights into the molecular mechanisms underlying AML progression. To bridge this knowledge gap, we established CD69 knockdown cell lines using two AML cell lines, K562 and KASUMI-1, through shRNA lentiviral transduction (**Figure 7B**). In the CCK-8 assay, we observed a significant reduction in cell viability in CD69 knockdown (sh-CD69) AML cell lines (**Figure 7C**). Furthermore, cell cycle analysis revealed a marked increase in the proportion of cells in the G2/M phase in the sh-CD69 AML cells (**Figure 7D**), suggesting that CD69 depletion may impair cell proliferation by inducing cell cycle arrest at the G2/M phase (**Figures 3D, E**, **7D**). Flow cytometric analysis was performed to assess the frequency of apoptosis in AML cells expressing either Scramble shRNA (Scr) or sh-CD69, Annexin V staining was used to identify and quantify apoptotic cells, the results indicated a higher proportion of apoptotic cells in the sh-CD69 group (**Figure 7E**). To further validate the results from the CCK-8 cell proliferation assay, we conducted an EdU incorporation experiment, which allowed us to visualize the proliferation status of AML cells by detecting newly synthesized DNA. As illustrated in **Figures 7F-I**, the EdU positivity rate decreased in the CD69 knockdown AML cells, indicating a reduction in newly synthesized DNA. Additionally, in colony formation assays, we found that the low expression of CD69 also significantly diminished the colony-forming ability of AML cells (**Figures 7L-O**). Notably, CD69 knockdown resulted in a significant increase in the frequency of CD11b and CD14 positive cells in K562 cells, as well as an increase in CD14 positive cells in KASUMI-1 cells. Furthermore, we observed that the KASUMI-1 cell line exhibited poor staining for CD11b during the immunophenotyping process, suggesting either reduced accessibility of the CD11b antigen on the cell membrane or overall low expression levels (**Figures 7H-L**). These findings highlight the potential role of CD69 in promoting AML cell survival and malignant proliferation, further supporting the role of CD69 in regulating cell survival and highlighting its potential as a therapeutic target in AML.

### *In vivo* efficacy of BTK-IN-8 treatment in MLL-AF9 leukemia mouse models

BTK (Bruton’s tyrosine kinase) is an essential tyrosine kinase primarily expressed in various immune cells. BTK inhibitors selectively suppress the kinase activity of BTK, a mechanism that effectively reduces the growth and spread of hematologic tumor cells (26). BTK-IN-8 demonstrates strong targeting activity against CD69 (27). The *in vivo* efficacy of BTK-IN-8 was evaluated using the MLL-AF9 leukemia mouse model, where two treatment arms were implemented: a control vehicle group and a BTK-IN-8 treatment group (4 mg/kg daily) (**Figure 7P**). Leukemia burden was assessed across multiple sites, including bone marrow, spleen, and peripheral blood, at several time points (D3, D6, D10, and D19). In addition, the survival rates of the mice were monitored and compared between the two groups. Flow cytometry analysis was used to quantify the percentage of GFP-positive leukemia cells in the bone marrow, spleen, and peripheral blood (**Figure 7Q**). In both the vehicle (Scr) and BTK-IN-8 treated groups, there was a significant reduction in GFP-positive leukemia cells in the peripheral blood and bone marrow following BTK-IN-8 administration. Specifically, at days D3, D6, D10, and D19, the percentage of GFP-positive cells in the drug treatment group was notably lower compared to the Scr group, suggesting effective clearance of leukemia cells (**Figure 7S**). Additionally, the spleen size of mice in the BTK-IN-8 treatment group was significantly reduced compared to the Scr group (**Figure 7R**), which further indicates the therapeutic potential of BTK-IN-8. The survival curves of the two groups clearly demonstrated an extended survival time in the BTK-IN-8 treated group, with a significant improvement in overall survival compared to the control (**Figure 7T**, P<0.01). These findings provide strong evidence supporting the efficacy of BTK-IN-8 as a potential therapeutic agent for MLL-AF9 leukemia.

## Discussion

In this study, we present the first comprehensive exploration of the relationship between prognosis-related gene signatures, overall survival, and potential target genes in AML (1, 5). Our findings highlight the limitations of traditional prognostic markers, such as FAB classification, bone marrow blasts (BmBlast), and peripheral blood blasts (PbBlast), in accurately assessing AML prognosis and determining optimal treatment timing. These conventional markers fail to account for the molecular heterogeneity of AML and lack the precision needed for personalized therapy, potentially leading to overtreatment or undertreatment of patients (28, 29). Given these challenges, our study underscores the urgent need for more robust, data-driven models that integrate clinical and molecular features for better risk stratification and therapeutic decision-making in AML.

Through a comprehensive single-cell RNA sequencing (scRNA-seq) analysis, we identified 621 malignant marker genes associated with AML. Consensus clustering of AML patients from the TCGA and GEO cohorts revealed significant enrichment in immune response and cell proliferation pathways in the high-risk group. Most existing AML prognostic models rely on subjective choices of algorithms, whereas the 101-machine-learning-algorithm framework provides an objective approach to identify the most suitable method for prognostic prediction in AML cohorts. To further refine our findings, we constructed and validated a prognostic model, MLAPS, using 101 machine learning algorithm combinations across four independent AML cohorts. Although the conventional FAB classification can guide the determination of different treatment strategies, it fails to account for the molecular heterogeneity of AML and lacks universal applicability in prognostic prediction. MLAPS effectively compensates for these shortcomings. MLAPS demonstrated superior predictive performance compared to common clinicopathological features and existing prognostic markers, highlighting its potential as a valuable tool for AML prognosis and risk stratification. In other solid tumors, the average highest C-index among 101 combination models often arises from combinations of two models with dimensionality reduction, which may offer better extrapolation potential (16, 17). However, in our research, the best-performing model is the RSF model, with the LASSO-RSF combination ranking second in terms of average C-index. One possible explanation is that LASSO, a linear regression method used for sparse feature selection, applies L1 regularization to shrink certain regression coefficients to zero, thereby selecting the most representative features. However, in the actual AML cohort data, LASSO may tend to select only a small number of variables (30, 31). If multiple features contribute similarly to the prognostic model, LASSO might retain only some of them, potentially excluding other informative features (30, 31). In the context of survival analysis for AML, some important features may be discarded due to regularized in LASSO, which could explain the performance drop in the LASSO-RSF combination. The RSF model demonstrated the highest C-index (concordance index, a key metric for evaluating the predictive accuracy of survival models, reflecting the consistency between predicted and observed survival rankings, with values ranging from 0.5 to 1.0, where higher values indicate stronger discriminatory ability), and it also exhibited robust predictive performance in other independent validation cohorts. Therefore, through an objective prognostic gene selection strategy and the training of 101 models, we identified RSF as the optimal base model for constructing MLAPS.

Interestingly, we observed that the high MLAPS group not only had a poorer prognosis but also exhibited a higher immune score, suggesting potential immune dysregulation within this cohort. Specifically, this implies a reduced abundance of natural killer (NK) cells and activated T cells in the tumor microenvironment, which could indicate the presence of immunosuppressive mechanisms contributing to the adverse prognosis (32, 33). This finding warrants further investigation into the specific immune pathways and regulatory factors involved, as such insights could inform future therapeutic strategies aimed at enhancing anti-tumor immunity in AML.

One of the key immune-related findings in our study was the identification of CD69 as a critical marker. CD69 is an early activation marker on various immune cells, playing a significant role in immune response regulation, inflammation, and lymphocyte tissue retention (34). While CD69 has been implicated in promoting anti-tumor immune responses in several solid tumors by regulating NK cell activity, it may also contribute to immune evasion by enhancing the suppressive function of regulatory T cells (Tregs), thereby limiting the activity of effector T cells (24, 35). In our MLAPS model, CD69 was ranked among the top variables in terms of importance, highlighting its potential relevance in AML prognosis and progression. Previous studies have reported the presence of CD69+ hematopoietic stem cell (HSC)-like leukemia subpopulations across various AML subtypes, associated with poor genetic profiles and clinical outcomes (25). However, our research provides a more in-depth analysis of CD69’s biological role in AML. We demonstrated that low expression of CD69 in AML cells leads to reduced proliferation and increased apoptosis, as evidenced by assays on colony formation, DNA replication, cell cycle progression, differentiation markers, and apoptotic activity. These findings suggest that CD69 could serve as a therapeutic target in AML. Furthermore, we explored the potential of BTK-IN-8, a novel anti-leukemia agent with CD69 inhibitory activity (26), which showed promising results in AML mouse models. However, further clinical trials are needed to confirm the efficacy of CD69-targeting therapies in AML patients.

Although this study incorporated large-scale, multi-center AML cohorts with strong representativeness and a certain degree of statistical robustness, several limitations remain. First, most publicly available or collaboratively obtained real-world or clinical trial AML cohorts lack systematic and detailed documentation of chemotherapy-related information, such as specific intensive chemotherapy regimens, dose intensity, number of treatment cycles, and tolerance to adverse effects. This limitation hinders precise validation of the model’s predictive performance under different treatment strategies. Second, the proposed MLAPS model was primarily validated in the overall population, and its applicability within treatment-stratified subgroups remains to be clarified. For example, whether the model’s high- and low-risk stratification can effectively distinguish prognostic differences in patients receiving conventional intensive chemotherapy, and whether the model retains comparable predictive value in patients treated with less-intensive regimens (e.g., Venetoclax combined with hypomethylating agents), are questions for which sufficient evidence is currently lacking. Future research, incorporating prospectively collected treatment-related data, will be essential to further assess the model’s generalizability and clinical utility across treatment contexts of varying intensity. In addition, this study revealed that CD69 expression in AML patients may have prognostic relevance; however, its biological and clinical significance under different therapeutic settings remains unclear. Specifically, in the context of intensive chemotherapy, it remains to be determined whether CD69 expression levels could serve as an additional biomarker to refine risk stratification or reflect differential sensitivity to chemotherapy. Likewise, under less-intensive regimens, whether CD69 expression retains independent discriminatory value, or instead exerts its influence only within particular molecular subtypes, is not yet established. Future studies integrating single-cell sequencing, functional experiments, and treatment cohort outcome analyses will be needed to systematically elucidate the role and clinical potential of CD69 in AML under diverse therapeutic settings.

While MLAPS shows significant clinical promise for predicting AML prognosis, several limitations in this study must be addressed. First, all datasets used were derived from publicly available AML databases, and the model’s validation should ideally be extended to prospective, multicenter studies to ensure its broader applicability. Additionally, certain clinical and molecular features in public datasets may be incomplete or unreliable, potentially impacting the accuracy and robustness of the model’s associations (36). These limitations highlight the need for more comprehensive validation to ensure the generalizability and accuracy of MLAPS across diverse clinical settings.

## Conclusion

Our comprehensive prognostic signature marks a substantial improvement over previous models by integrating machine learning algorithms with multiple independent validation cohorts. The robust performance and wide applicability of our model across diverse datasets highlight its reliability and strength as a clinical tool. Once validated in clinical settings, MLAPS has the potential to significantly enhance treatment strategies by pinpointing patients at risk of aggressive disease progression and poor outcomes under standard therapies. Additionally, we identified CD69 as a key molecule in AML progression, with promising potential as a therapeutic target for AML. Taken together, MLAPS offers a highly adaptable platform that can enhance risk stratification, prognostic accuracy, and therapeutic target identification, ultimately aiding in the improved clinical management of AML patients.

## Data Availability

The datasets presented in this study can be found in online repositories. The names of the repository/repositories and accession number(s) can be found in the article/**Supplementary Material**.
