# Supplementary material for: Integrated machine learning-based establishment of a prognostic model in multicenter cohorts for acute myeloid leukemia
Source: Front Oncol. 2025 Oct 14;15:1649594. doi: 10.3389/fonc.2025.1649594 (PMC12560054; doi:10.3389/fonc.2025.1649594)
Supplement: Supplementary file 1 [file Image1.pdf]

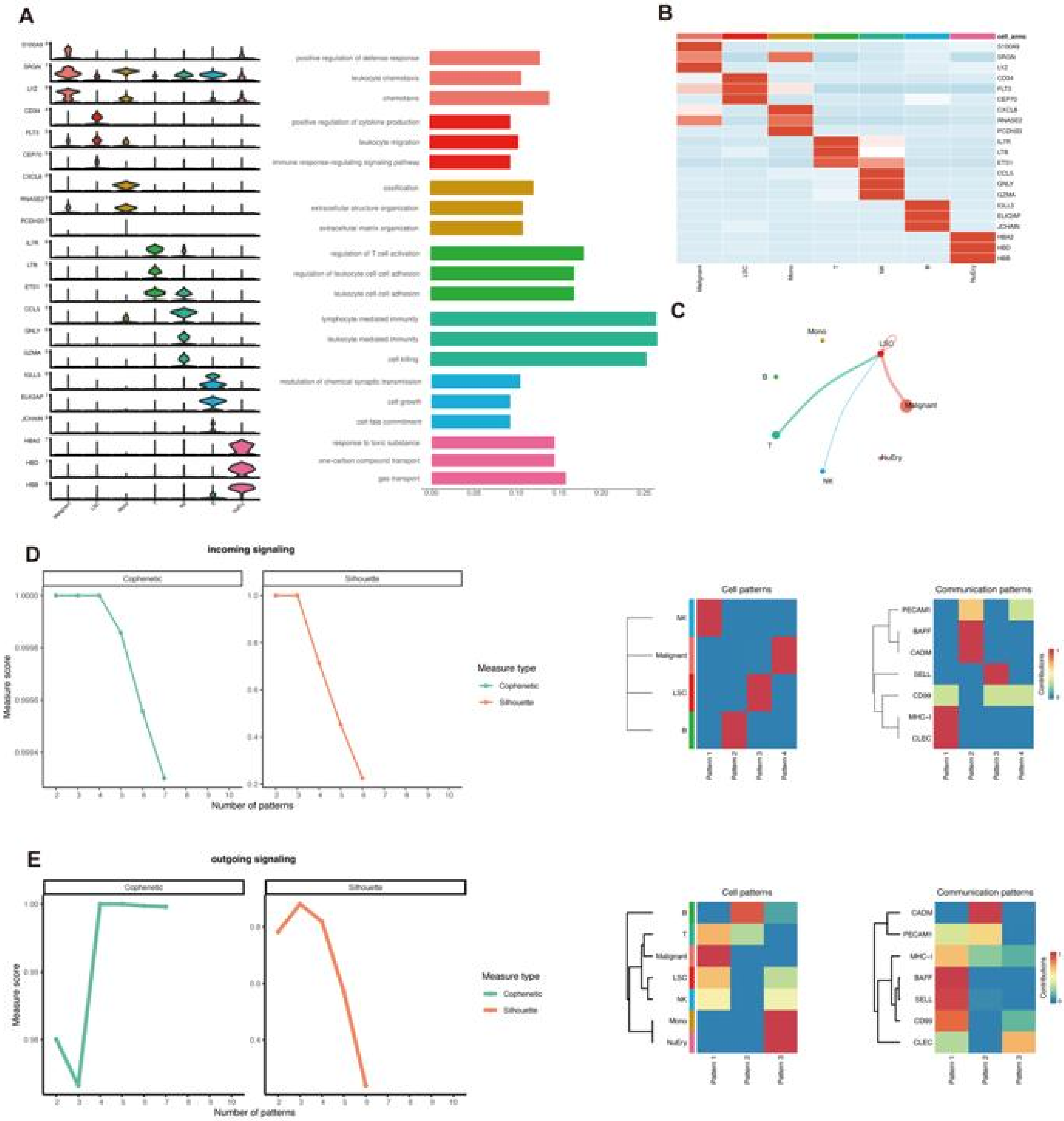
A
B
C
D
E
positive regulation of defense response
leukocyte chemotaxis
chemotaxis
positive regulation of cytokine production
leukocyte migration
immune response-regulating signaling pathway
ossification
extracellular structure organization
extracellular matrix organization
regulation of T cell activation
regulation of leukocyte cell-cell adhesion
leukocyte cell-cell adhesion
lymphocyte mediated immunity
leukocyte mediated immunity
cell killing
modulation of chemical synaptic transmission
cell growth
cell fate commitment
response to toxic substance
one-carbon compound transport
gas transport
cell_anno
Mono
LSC
B
Malignant
T
NK
NuEry
incoming signaling
outgoing signaling
Cophenetic
Silhouette
Measure score
Number of patterns
Measure type
Cell patterns
Communication patterns
Contributions
PECAM1
BAFF
CADM
SELL
CD99
MHC-I
CLEC
Pattern 1
Pattern 2
Pattern 3
Pattern 4

FigS1. Bone marrow microenvironment landscape in AML

A. The top DEGs and GO biological processes of each cell cluster.

B. The heatmap shows top DEGs average expression in each cell cluster.

C. The chord plot presents the inferred intercellular communication network between AML cells, NK cells, and T cells.

D-E. The line chart illustrates the relationship between Measure score and Number of patterns, while the heatmap illustrates the patterns of cell communication. D: incoming signaling, and E: outgoing signaling.

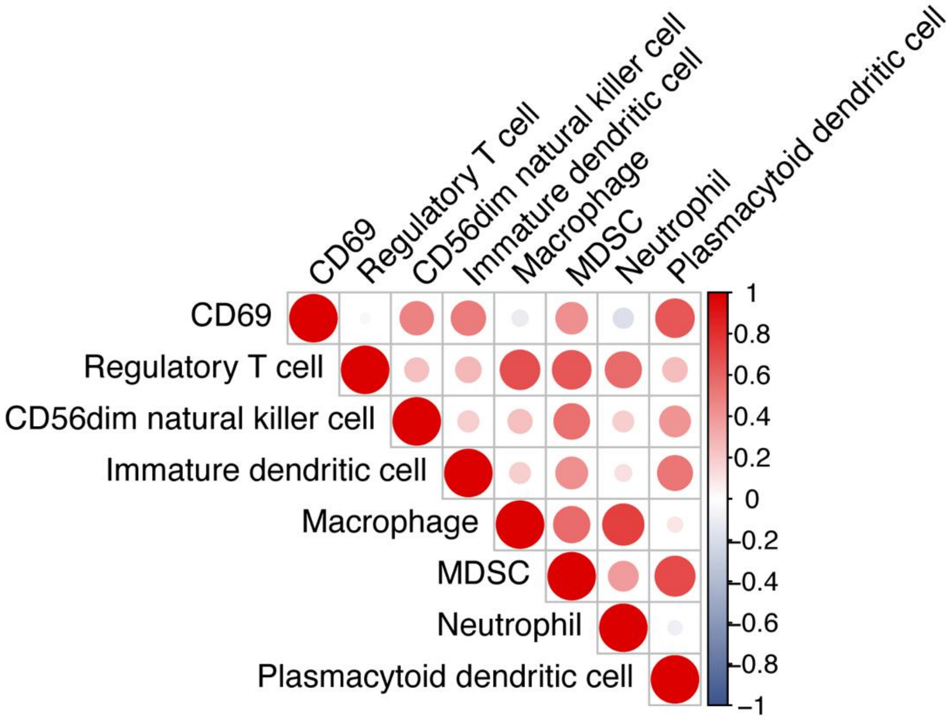

FigS2. Correlation between CD69 and pro-tumor immune cell infiltration in AML

Dot size represents the absolute value of the correlation; red indicates a positive correlation, and blue indicates a negative correlation.
